# Recombinant human mitsugumin 53: a potential therapeutic agent for multiple diseases

**DOI:** 10.3389/fphar.2025.1625232

**Published:** 2025-09-22

**Authors:** Junfeng Li, Jingjing Shen, Tian Li, Liqun Wang

**Affiliations:** ^1^ Department of Oncology of Zaozhuang Hospital of Zaozhuang Mining Group, Zaozhuang, Shandong, China; ^2^ Department of Gynaecology and Obstetrics of People’s Hospital of Xuecheng, Zaozhuang, Shandong, China; ^3^ Basic Medicine Research Innovation Center for Cardiometabolic Diseases, Ministry of Education, Luzhou, Sichuan, China; ^4^ Luzhou Municipal Key Laboratory of Thrombosis and Vascular Biology, Luzhou, Sichuan, China; ^5^ Laboratory for Cardiovascular Pharmacology, Department of Pharmacology, School of Pharmacy, Southwest Medical University, Luzhou, Sichuan, China; ^6^ Metabolic Vascular Disease Key Laboratory of Sichuan Province, The Affiliated Hospital, Southwest Medical University, Luzhou, Sichuan, China

**Keywords:** recombinant human MG53, cardiovascular diseases, acute lung injury, kidney injury, tumor

## Abstract

Mitsugumin 53 (MG53), a member of the tripartite motif family proteins, is predominantly expressed in skeletal and cardiac muscles. Endogenous MG53 was first identified as a crucial component of the sarcolemmal membrane repair machinery. In cardiac muscles, MG53 prevented against ischemia/reperfusion-induced myocardial injury. Recent research has demonstrated that native MG53 is present in and performs significant functions within various non-striated muscle tissues, including lung epithelial cells, the inner cortex of the kidney, macrophages, aortic valves, corneal epithelial cells, as well as in the tear film and aqueous humor. These studies indicate a potential application of recombinant human MG53 (rhMG53) in various disease models. To date, an increasing number of studies have confirmed that rhMG53 exhibits broad protective effects in skeletal muscle injury, cardiovascular diseases, and other multiple-organ injuries. In this review, we clarify the therapeutic functions of rhMG53 and the molecular mechanisms involved in multiple organ injuries, emphasizing the development of rhMG53 protein as a potential therapeutic agent for treating a range of diseases.

## 1 Introduction

Mitsugumin 53 (MG53), which belongs to tripartite motif (TRIM) protein family, is also known as TRIM72. With a molecular weight of 53 kD, the human MG53 protein consists of 477 amino acids. MG53 is composed of a typical TRIM structure at the N-terminus and a PRY-SPRY domain at the C-terminus. The TRIM structure includes a RING domain, one or two B-box domains, and a coiled-coil domain (Cai et al., 2009; Zhong et al., 2021; Li et al., 2021b). The RING domain is a zinc-finger motif that contains a Cys3HisCys4 amino acid sequence, enabling it to bind two zinc cations (Li et al., 2021b; Zhang et al., 2017). Importantly, the RING domain functions as an E3 ubiquitin ligase, catalyzing the ubiquitination and degradation of multiple target proteins. A critical cysteine residue (C14) within the RING domain is essential for MG53’s activity as an E3 ubiquitin ligase (Hatakeyama, 2017). The B-box domain is another zinc-binding motif, and its specific function may be associated with innate immune system defense, cell membrane repair, and wound healing (Ozato et al., 2008). The coiled-coil domain regulates homo- and hetero-oligomerization among TRIM family proteins (Zhang et al., 2017; Ozato et al., 2008), while the PRY-SPRY domain is believed to facilitate selective binding with target proteins (Alloush and Weisleder, 2013).

MG53 is predominantly expressed in striated muscles (cardiac and skeletal muscles). Notably, MG53 is highly abundant in the cardiac and skeletal muscles of mice and rats, while its expression is significantly lower in the human heart (Tan et al., 2016). Cai et al. has firstly reported that the MG53 in skeletal muscles can facilitate the repair of acute plasma membrane damage as an essential component in the membrane repair machinery. Mice with gene knockout of MG53 (*Mg53* ^*−/−*^) exhibit a loss of membrane repair function in striated muscle cells, leading to progressive skeletal muscle pathology (Cai et al., 2009). Additionally, MG53 in skeletal muscles negatively regulates myogenesis by ubiquitin-mediated degradation of insulin receptor substrate-1 (IRS-1) and focal adhesion kinase (FAK) (Yi et al., 2013; Nguyen et al., 2014). In cardiac muscles, MG53 plays an important protective role against ischemia/reperfusion (I/R)-mediated myocardial injury by participating in ischemic preconditioning and postconditioning (Wang et al., 2010; Cao et al., 2010; Zhang et al., 2011).

Furthermore, native MG53 is also found in lung epithelial cells (Jia et al., 2014; Kim et al., 2014), the inner cortex of the kidney (Duann et al., 2015; Li et al., 2022a), human macrophages (Sermersheim et al., 2020), corneal epithelial cells (Chandler et al., 2019), tear film and aqueous humor (Chandler et al., 2019), albeit at much lower expression levels compared to striated muscles. Although the expression level of MG53 in the lung epithelial cells is much lower (approximately 5% of the expression in skeletal muscle), *Mg53* ^*−/−*^ mice display defective alveolar structure and show more susceptible to I/R- and overventilation-induced lung injury when compared with wide type mice (Jia et al., 2014; Kim et al., 2014). MG53 is also present in the renal proximal tubular epithelial cells at a low but significant concentration (approximately 2.5% of the expression in skeletal muscles), which prevents against nephrotoxin- and IR-induced acute kidney injury through participating in repair of injured proximal tubular epithelial cells (Duann et al., 2015; Li et al., 2022a). The expression level of MG53 in human macrophages is over 400 times lower than that in skeletal muscles, where it suppresses inflammation by regulating ryanodine receptor-mediated intracellular calcium signaling (Sermersheim et al., 2020). Native MG53 can also be detected in corneal epithelial cells, tear film, and aqueous humor, indicating its potential role in corneal homeostasis (Chandler et al., 2019).

More interestingly, although native MG53 is absent in various tissues, MG53, as a myokine/cardiokine, can be secreted into the bloodstream from cardiac and skeletal muscles (Wu et al., 2019), participating in the regulation of multiple physiological and pathological processes. This suggests that therapies utilizing MG53 are less likely to provoke an immune system reaction. Furthermore, an increasing number of studies have confirmed that recombinant human MG53 (rhMG53) exhibits broad protective effects in various disease models. In this review, we clarify the therapeutic functions of rhMG53 and the molecular mechanisms involved in multiple organ injuries (Table 1), emphasizing the development of rhMG53 protein as a potential therapeutic agent for treating various diseases.

**TABLE 1:** The therapeutic applications of rhMG53.

Target organs	Therapeutic effects of rhMG53 and the molecular mechanisms involved
1. Heart	1.1. Prevent against angioplasty-induced MI in a porcine model via promoting cardiomyocyte survival (Liu et al., 2015). 1.2. Prevent against I/R-induced oxidative stress in cardiomyocytes via preserving mitochondrial integrity (Gumpper-Fedus et al., 2022). 1.3. Inhibit the progression of valvular heart disease through mediating membrane repair of valve interstitial cells and reducing the activation of the TGF-β signaling pathway (Adesanya et al., 2019).
2. Lung	2.1. Ameliorate I/R-, PPE- and LPS-induced lung injuries by repairing lung epithelial cell membranes and reducing pro-inflammatory factors (Jia et al., 2019). 2.2. Prevent against ventilation-induced lung injuries by improving the resilience of alveolar epithelial cells to membrane wounding (Nagre et al., 2018). 2.3. Inhibit nitrogen mustard-induced lung injuries by restoring arterial blood oxygen levels, improving dynamic lung compliance, and decreasing airway resistance (Li et al., 2022b). 2.4. Ameliorate bleomycin-induced lung fibrosis through inhibiting p53 activation and reducing cell apoptosis (Cong et al., 2020). 2.5. Prevent against a lethal respiratory infection caused by the H1N1 virus via reducing cytokine storms and inhibiting pyroptosis (Kenney et al., 2021).
3. Kidney	3.1. Ameliorate cisplatin- and contrast-induced AKI by reducing tubular cell membrane damage and apoptosis (Duann et al., 2015; Liu et al., 2020). 3.2. Protect the kidney by involving local polymerase I and transcript release factor after severe burn injury (Wu et al., 2013). 3.3. Protect against unilateral ureteral obstruction-induced kidney fibrosis by inhibiting the activation of NF-κB signaling and reducing pro-inflammatory programs (Li et al., 2022a).
4. Liver	4.1. Prevent against IR-induced liver injuries through inhibiting hepatic oxidative stress and hepatocyte death (Yao et al., 2017). 4.2. Prevent against drug-induced liver injuries by directly entering hepatocytes, improving membrane integrity and increasing hepatocyte survival (Han et al., 2022).
5. Skeletal muscles	5.1. Protect against chemical, mechanical, or UV-induced muscle damages through improving the membrane repair capacity of skeletal muscle fibers (Weisleder et al., 2012). 5.2. Protect against both acute and chronic muscle injuries induced by eccentric contractions not only by enhancing the repair of muscle sarcolemma injuries but also by promoting the proliferation of muscle satellite cells (Bian et al., 2019). 5.3. Protect against I/R-induced injures in mice through improving muscle structure (Zhu et al., 2015). 5.4. Treat LGMD2B by enhancing the membrane integrity of skeletal muscle fibers (Gushchina et al., 2017).
6. Cornea	6.1. Prevent against alkaline-induced corneal injuries by enhancing promoting the migration of corneal fibroblasts and inhibiting TGF-β-mediated fibrotic remodeling (Chandler, et al., 2019; Guo et al., 2021). 6.2. Prevent against alkaline-induced corneal neovascularization by inhibiting FAK-paxillin signalling activation and decreasing FA turnover and tip cell formation (Dong et al., 2021; Yuan et al., 2025).
7. Tumor	7.1. Enhance the susceptibility of NSCLC cells to cisplatin-induced apoptosis through facilitating the nuclear translocation of G3BP2 (Li et al., 2021a). 7.2. Inhibit tumor growth in a mouse SW620/AD300 xenograft model (Gupta et al., 2022).

## 2 The cardioprotective effects of rhMG53

Cardiovascular diseases have been identified as the leading cause of mortality worldwide, imposing significant physical and economic burdens on both patients and the public health system as a whole (Timmis et al., 2024). This category of disease encompasses a range of conditions affecting the heart and blood vessels, including angina pectoris (AP), coronary heart disease (CHD), myocardial infarction (MI), heart failure (HF), hypertension, congenital heart defects, valvular disorders, coronary artery disease (CAD), peripheral artery disease (PAD), venous diseases, and cerebrovascular accidents (strokes). Recently, serum MG53 has been identified as a novel biomarker for diagnosing CAD and indicating its severity (Xie et al., 2020; Xie et al., 2020), highlighting the critical roles of MG53 in cardiovascular diseases. In addition, endogenous MG53 has been demonstrated to prevent against cardiac I/R injury (Wang et al., 2010; Cao et al., 2010; Zhang et al., 2011), valvular heart disease (Adesanya et al., 2019), hypertrophic cardiomyopathy (Liu et al., 2019), and heart failure (Wang et al., 2021), providing evidence for the use of rhMG53 in the treatment of cardiovascular disease.

In a study utilizing a porcine model of angioplasty-induced MI, Liu et al. demonstrated that the intravenous administration of rhMG53, either before or after the onset of ischemia, resulted in a reduction of infarct size and troponin I release. Subsequent echocardiographic and histological evaluations indicated that the cardioprotective effects of rhMG53 observed in the acute phase of MI were associated with long-term enhancements in cardiac structure and function, as assessed 4 weeks following the surgical intervention (Liu et al., 2015). A subsequent study showed that rhMG53 translocated to the mitochondria following ischemic injury, both *in vivo* and *in vitro*. This translocation resulted in a reduction in superoxide production and an inhibition of mitophagy, suggesting that the interactions between MG53 and mitochondria may represent a novel mechanism through which rhMG53 treatment mitigates cardiomyocyte injury and preserves cardiac function following ischemic events (Gumpper-Fedus et al., 2022). Moreover, research has demonstrated that intravenously administered rhMG53 accumulates at the site of injury in the aortic valve. As a membrane repair factor, exogenous rhMG53 is expected to protect valvular interstitial cells from membrane damage and to inhibit the progression of valvular heart disease (Adesanya et al., 2019). Additionally, rhMG53 has been shown to attenuate the fibrotic process in valvular interstitial cells by modulating the TGF-β signaling pathway (Adesanya et al., 2019). These findings suggest that rhMG53 may have significant potential for medical applications in the prevention and treatment of valvular heart disease.

## 3 A potential role of rhMG53 in treatment of lung injury

Previous studies have shown that endogenous MG53 is present in alveolar epithelial cells and that *Mg53* ^*−/−*^ mice exhibit abnormal lung structure and function under basal conditions (Jia et al., 2014; Kim et al., 2014). The expression of MG53 in the lung significantly increased following tidal volume (IV), HCL, and bleomycin-induced lung injuries (Cong et al., 2020). Furthermore, the bioinformatics analysis also identified elevated MG53 expression in airway epithelial cells from patients with severe asthma (Voraphani et al., 2014), in type II alveolar epithelial (ATII) cells from surfactant protein C-deficient mice (Glasser et al., 2013), in human airway epithelial Calu-3 cells infected with H5N1 virus and in type I alveolar epithelial (ATI) and ATII cells from human idiopathic pulmonary fibrosis (IPF) lung (Cong et al., 2020). All of these data suggest that MG53 may function as an injury-responsive protein and could play a protective role in a wide range of lung injuries.

Therefore, a series of studies have investigated whether the systemic application of rhMG53 can prevent against acute and chronic lung injuries. Using the rodent animal models, Jia et al. demonstrated that both intravenous and intra tracheal delivery of rhMG53 effectively mitigated lung I/R injury, lipopolysaccharide (LPS) exposure, and porcine pancreatic elastase (PPE)-induced emphysema (Jia et al., 2019). Additionally, repetitive administration of rhMG53 has shown beneficial effects on the structural and functional changes in the lung associated with emphysema. Mechanically, the repair of lung epithelial cell membranes and the reduction of pro-inflammatory factors may contribute to the pulmonary protective effects of rhMG53 (Jia et al., 2019). Inhaled rhMG53 has also been shown to directly enter alveolar epithelial cells in a cholesterol-dependent manner, enhancing cell resilience to membrane damage and consequently preventing against ventilation-induced lung injury (Nagre et al., 2018). Li et al. further identified that treatment of wild-type mice with exogenous rhMG53 inhibits nitrogen mustard-induced lung injury by restoring arterial blood oxygen levels, improving dynamic lung compliance, and decreasing airway resistance (Li et al., 2022b). Interestingly, intraperitoneal application of rhMG53 following bleomycin treatment on days 7–11 significantly ameliorated bleomycin-induced lung fibrosis through repairing the membrane injury of ATIIs, leading to the inhibition of p53 activation and a reduction in cell apoptosis (Cong et al., 2020). These results support the therapeutic potential of rhMG53 once injury-induced fibrosis has been established, as observed in patients with IPF.

As one of the world’s greatest public health challenges, respiratory virus infections, including influenza, rank among the top ten causes of human mortality (Iuliano et al., 2018). Recently, Kenney et al. demonstrated that administration of rhMG53 following a lethal respiratory infection caused by the influenza virus (H1N1) protected against virus-induced lung injury via mitigation of cytokine storm and inhibition of pyroptosis, leading to a nearly absolute survival benefit (Kenney et al., 2021). These findings provide evidence supporting the further clinical development of rhMG53 as a biologic treatment for inflammation-driven infectious diseases.

## 4 The protective effects of rhMG53 on kidney injury

Renal proximal tubular epithelial cells (PTECs) are highly susceptible to membrane damage under stress conditions such as chemotherapy, I/R, nephrotoxin, or sepsis, all of which are involved in the development of acute kidney injury (AKI) and chronic kidney disease (CKD) (Ke et al., 2023). Although the native MG53 protein is primarily expressed in striated muscle, studies have demonstrated that MG53 is also present in the inner cortex of the kidney (Duann et al., 2015; Li et al., 2022a). Moreover, both endogenous MG53 and rhMG53 can target injury sites on PTECs to facilitate repair of cell membrane (Duann et al., 2015; Liu et al., 2020), indicating that rhMG53 may display significant therapeutic potential for kidney disease through protecting PTECs.

In animal studies, intravenous delivery of rhMG53 inhibits cisplatin-induced AKI without affecting its tumor suppressor efficacy (Duann et al., 2015). Liu et al. also reported that pretreatment of rats with rhMG53 protected against contrast-induced AKI by reducing tubular cell membrane damage and apoptosis (Liu et al., 2020). Utilizing a scalding model of 30% of total body surface area, another group investigated the effects of rhMG53 on the protection of kidneys after severe burn injury and the results demonstrated that intravenous injection of rhMG53 reduced the mortality and the histological alternation of renal tubular epithelial cells after burn damage (Wu et al., 2013). Moreover, Li et al. further confirmed that rhMG53 protected against unilateral ureteral obstruction-induced kidney fibrosis by inhibiting the phosphorylation and activation of NF-κB signaling, reducing p65 nuclear translocation, and blocking activation of pro-inflammatory programs (Li et al., 2022a). Taken together, all of the studies suggest that pharmacologic administration of rhMG53 might be a promising approach for the treatment of AKI and CKD.

## 5 The effects of rhMG53 on liver protection

Liver I/R is characterized by a series of pathological insults resulting from hypoxia and re-oxidation processes associated with the interruption and subsequent restoration of blood flow, which leads to significant hepatic damage due to oxidative stress, inflammation, and mitochondrial dysfunction (de Oliveira and Goncalves, 2025). The I/R injury has been demonstrated to be a critical challenge in liver transplantation, resection, and trauma surgeries. Yao et al. demonstrated that the administration of exogenous rhMG53 effectively interacted with dysferlin, thereby protecting against IR-induced damage to the hepatocyte membrane. Consequently, this interaction reduced hepatic oxidative stress and hepatocyte death, ultimately preventing severe hepatic I/R injury (Yao et al., 2017). This study indicates the potential utility of rhMG53 for the treatment of diseases associated with liver I/R damage.

Drug-induced liver injury is a leading cause of acute liver failure, resulting in high morbidity and mortality rates and the treatment of drug-induced liver injury remains a challenge. Han et al. showed that liver injury caused by acetaminophen tetracycline, concanavalin A, carbon tetrachloride, or thioacetamide in mice can be both prophylactically and therapeutically treated with the systemic application of rhMG53 protein. Mechanistically, extracellular rhMG53 directly entered hepatocytes, improving membrane integrity and functioning as an E3-ligase to mitigate receptor-interacting protein kinase 3 (RIPK3)-mediated phosphorylation and membrane translocation of mixed lineage kinase domain-like protein (MLKL), which ultimately increased the survival of the cells (Han et al., 2022). These results suggest that rhMG53 may be a potential treatment option for patients with drug-induced liver injury.

## 6 The role of rhMG53 in repair and regeneration of skeletal muscles

Skeletal muscle cells consistently experience significant membrane stress in response to the routine contraction and relaxation of skeletal muscles, leading to sarcolemma injury under normal physiological conditions in an average life span. A diminished capacity for membrane repair has been associated with various diseases, highlighting the critical importance of acute muscle membrane repair (McNeil and Khakee, 1992). Endogenous MG53 has been identified as a key element of the plasma membrane repair mechanism, which facilitates repair of acute membrane damage in an oxidation-dependent manner (Cai et al., 2009; Alloush and Weisleder, 2013; McNeil, 2009). In addition to its intracellular functions, MG53 can detect signals released by injured cell membranes. This allows rhMG53 to repair membrane damage when introduced into the extracellular space.

Extracellular rhMG53 can detect external signals during membrane disruptions and enhance membrane resealing in cultured muscle cells *in vitro*, offering protection against chemical, mechanical, or UV-induced damage (Weisleder et al., 2012). The administration of rhMG53 via various methods, including intramuscular (IM), intravenous (IV), and subcutaneous (SC) injections, can improve the membrane repair capacity of skeletal muscle fibers and mitigate the pathology associated with muscular dystrophy in the mdx mouse model (Weisleder et al., 2012). Additionally, the intravenous administration of rhMG53 protects against both acute and chronic muscle injury induced by eccentric contractions in mice, not only by enhancing the repair of muscle sarcolemma injuries but also by promoting the proliferation of muscle satellite cells (Bian et al., 2019). Furthermore, rhMG53 applied intravenously protects against I/R-induced skeletal muscle injures in mice rather than in rats (Zhu et al., 2015). This difference is attributed to the significantly higher plasma levels of endogenous MG53 in rats, which render rat muscle less responsive to the therapeutic effects of rhMG53 (Zhu et al., 2015).

Limb girdle muscular dystrophy type 2B (LGMD2B) and other dysferlinopathies are degenerative muscle diseases caused by mutations in the dysferlin gene, and they currently have limited treatment options (Barthelemy et al., 2011; Poudel et al., 2024). Recently, rhMG53 has been shown to enhance the membrane integrity of skeletal muscle fibers, independent of the traditional dysferlin-mediated and calcium -associated pathways (Gushchina et al., 2017). This suggests that rhMG53 may compensate for the inherent membrane repair defects observed in dysferlin-deficient mouse models, indicating its potential therapeutic benefits for patients with LGMD2B.

## 7 rhMG53-mediated repair in cornea injury

The cornea is the outermost layer of the eye, crucial for light transmission and protecting the internal structures of the eye. Being exposed to the external environment makes the cornea susceptible to injuries and infections. The high density of nerve endings in the cornea means that persistent wounds can be quite painful, and any delays in healing may increase the risk of scarring and vision loss.

The endogenous MG53 protein has also been identified in the corneal epithelium, tear film, and aqueous humor, indicating its potential role in maintaining corneal homeostasis (Chandler, et al., 2019). rhMG53 has been shown to prevent against alkaline-induced corneal injury by enhancing re-epithelialization while simultaneously decreasing post-injury fibrosis and vascularization (Chandler, et al., 2019; Guo et al., 2021; Wang et al., 2020). Mechanistically, rhMG53 protects the corneal epithelia cell from mechanical injury, promotes the migration of corneal fibroblasts and inhibits TGF-β-mediated fibrotic remodeling (Chandler, et al., 2019; Guo et al., 2021). Moreover, our research also demonstrated that rhMG53 enters endothelial cells via caveolae-dependent and clathrin-dependent endocytosis, and then reduces FAK-paxillin signalling activation, leading to decreased FA turnover and tip cell formation, ultimately inhibiting endothelial cell spreading, migration and tube formation, which contribute to the reduction of alkaline injury-induced corneal neovascularization (Dong et al., 2021; Yuan et al., 2025).

## 8 Anti-tumor effects of rhMG53

The relationship between endogenous MG53 and tumorigenesis and progression has been demonstrated. Previous studies have revealed that endogenous MG53 exerts inhibitory effects on the growth and metastasis of lung tumors (Li et al., 2021a), the malignant progression of hepatocellular carcinoma (Ma et al., 2022), colorectal cancer (Fang et al., 2023), pancreatic ductal adenocarcinoma (Wang et al., 2024), and the progression of tongue cancer cells (Yin et al., 2019). Therefore, the potential anti-tumor roles of rhMG53 were further investigated. The rhMG53 protein can penetrate non-small cell lung cancer (NSCLC) cells, facilitating the nuclear translocation of G3BP2 and inhibiting arsenic trioxide-induced stress granule formation (Li et al., 2021a). Additionally, it enhances the susceptibility of NSCLC cells to apoptosis when treated with cisplatin (Li et al., 2021a). Furthermore, in a mouse SW620/AD300 xenograft model, the administration of rhMG53 in combination with doxorubicin significantly reduced tumor growth without causing noticeable weight loss or hematological toxicity in the animals (Gupta et al., 2022). Recent studies strongly indicate that rhMG53 yields a significant therapeutic potential for tumors. However, the specific mechanisms by which it exerts its anti-tumor effects remain not fully illustrated.

## 9 Conclusions and prospects

**FIGURE 1:**
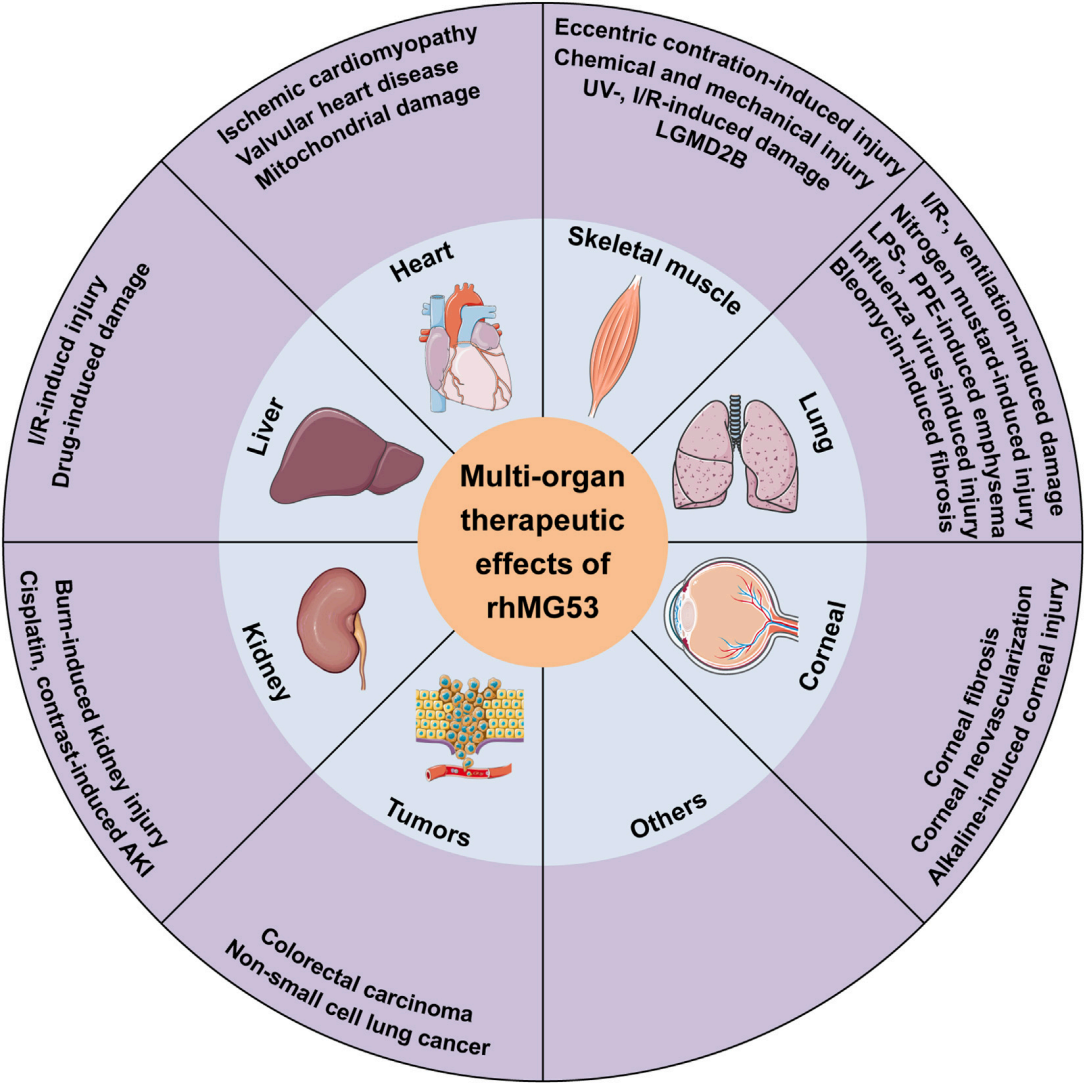
Multi-organ protective effects of rhMG53 on various diseases. rhMG53 has been demonstrated to prevent against skeletal muscle injuries, cardiovascular diseases, acute and chronic lung injuries, liver injuries, AKI, CKD, corneal injuries and tumors.

Numerous studies have recognized endogenous MG53 as a critical component in the process of cell membrane repair, with potential therapeutic applications for various injuries, including myocardial infarction, muscular dystrophy, and damage to non-muscular organs such as lungs, skin, and cornea. Recently, an increasing number of studies have explored the clinical application of rhMG53 for the treatment of various diseases in recent years. The results indicate that rhMG53 can prevent against skeletal muscle injuries, cardiovascular diseases, acute and chronic lung injuries, liver injuries, AKI, CKD, corneal injuries and tumors (Figure 1) (Table 1). Although current research on the functionality of rhMG53 predominantly relies on animal models, the findings also highlight the therapeutic value of rhMG53 protein as a potential agent to repair multiple-organ injuries.

More importantly, the safety and toxicity of rhMG53 have been thoroughly evaluated. Studies revealed that repeated intravenous administration of 1 mg/mL rhMG53 did not produce any adverse effects in rodents and dogs. Due to the higher renal clearance rate of rhMG53, its delivery effectively targets organs for therapeutic benefits without leading to accumulation. Furthermore, the maximum tolerated dose of rhMG53 in animal studies was established, demonstrating that repeated intravenous injections of up to 40 mg/mL, which was 40 times higher than the therapeutic dose, did not significantly affect metabolic function or cause systemic lethal toxicity. However, in recent years, the effects of MG53 on diabetes have gradually been elucidated, but the findings remain contentious (Yuan et al., 2025). Some studies suggest that the expression of MG53 is increased in metabolic disorders and that muscle-specific upregulation of MG53 is sufficient to induce whole-body insulin resistance and metabolic syndrome (Song et al., 2013). Additionally, as a myokine/cardiokine, circulating MG53 and rhMG53 have been shown to trigger insulin resistance by binding to the extracellular domain of the insulin receptor, thereby allosterically inhibiting insulin signaling (Wu et al., 2019). Conversely, other studies have reported contradictory findings. Specifically, no significant changes in MG53 expression in striated muscles or serum have been observed in diabetic models (Yi et al., 2013). Furthermore, sustained elevation of MG53 levels in serum or systemic administration of rhMG53 has shown no impact on metabolic function (Bian et al., 2019). Therefore, the effects of MG53 and rhMG53 on metabolic diseases, as well as the underlying molecular mechanisms, require further investigation, thereby providing a theoretical foundation for the treatment of metabolic disorders and the application of rhMG53.
